# Stereotactic radiosurgery, a potential alternative treatment for pulmonary metastases from osteosarcoma

**DOI:** 10.3892/ijo.2014.2295

**Published:** 2014-02-10

**Authors:** WENXI YU, LINA TANG, FENG LIN, DAKE LI, JUN WANG, YAO YANG, ZAN SHEN

**Affiliations:** 1 Department of Oncology, Affiliated Sixth People’s Hospital, Shanghai Jiaotong University, Xuhui, 200233 Shanghai;; 2 Department of Gynecology and Obstetrics, Jiangsu Province Hospital of TCM, Affiliated Hospital of Nanjing University of TCM, 210029 Nanjing;; 3 Department of First General Surgery, People’s Hospital of Ma An Shan, Ma An Shan, Anhui, P.R. China

**Keywords:** osteosarcoma, pulmonary metastasis, radiosurgery, gamma knife

## Abstract

Stereotactic radiosurgery (SRS), such as body gamma knife, was reported to achieve excellent rates of local disease control with limited toxicity in many cases of primary or secondary pulmonary tumor, except osteosarcoma. To confirm the value of SRS in pulmonary metastases from osteosarcoma, we reviewed the experience from our institution (Department of Oncology, Affiliated Sixth People’s Hospital, Shanghai) and compared the efficiency of SRS with that of surgical resection. From January 2005 to December 2012, we carried out a retrospective investigation of 58 patients (age, 8–59 years; mean, 25.2 years) who were diagnosed with non-metastatic osteosarcoma of the extremity and later developed pulmonary metastasis during the period of adjuvant chemotherapy or follow-up. Among them, 27 patients were treated by SRS using the body gamma-knife system. A total dose of 50 Gy was delivered at 5 Gy/fraction to the 50% isodose line covering the planning target volume, whereas a total dose of 70 Gy was delivered at 7 Gy/fraction to the gross target volume. The other 31 patients were treated by surgical resection. Two-year progression-free survival rate, two-year survival rate, median time of PRPFS (post-relapse progress-free survival) and PROS (post-relapse overall survival) in SRS group were parallel to that in surgical group. All 27 patients tolerated gamma knife radiosurgery well while only 9 patients had grades 1–2 pneumonitis. We believe SRS, compared with surgical resection, is effective and safe in treating pulmonary metastasis from osteosarcoma, especially for those patients who were medically unfit for a resection or who refused surgery.

## Introduction

Osteosarcoma (OS) is the most common malignant primary bone tumor in childhood (1), with an age-standardized incidence of ∼5 per million per year in America (2). It has a high propensity to metastasize to the lungs, and the prognosis for localized extremity OS treated with surgery alone is poor (<20% 2-year survival) (3). Despite improvements in multidisciplinary treatment, 30% of patients with localized disease and 80% with metastatic disease at diagnosis will relapse (4,5), even if they have received large dose of adjuvant/neoadjuvant chemotherapy. Failure of standard multimodal therapy for the OS is associated with a very poor prognosis. Surgical resection has been shown to prolong survival among patients with pulmonary metastases (6,7), including soft tissue sarcoma (7,8), and similar results are obtained for lung metastases from OS (9–16). China is a large country consisting of various people with different cultural backgrounds, and some patients are unwilling to undergo operation because of their traditional cultural custom, which limits the use of surgical resection. Besides, the efficacy of second-line chemotherapy still remains controversial while many investigators (13,14) have reported that outcome of patients suffered from relapsed OS treated only by surgery or by surgery combined with second-line chemotherapy was almost the same. Thus, development of novel techniques for OS patients with pulmonary metastasis is highly desired.

Stereotactic radiosurgery (SRS), such as gamma knife, is defined as the delivery of external beam radiation treatment technique by the use of several precisely aimed, highly focused beams targeted to ablate a specific lesion with a fixed, or virtual, stereotactic frame. Given its excellent rates of local disease control with limited toxicity to surrounding tissues, it has increasingly been accepted in the management of intracranial neoplasms, and interest has developed in noncranial applications. Obvious opportunities included tumors of the spine, lung, pancreas and prostate (17–20). Many cases of primary or secondary pulmonary tumors (17, 21–24), include pulmonary metastasis from soft tissue sarcoma (24,25), SRS offers a very effective treatment option without significant complications, especially in medically-impaired patients who are not surgical candidates or refuse surgery.

SRS-induced lung injury, or radiation pneumonitis, is usually related with tumor size and tumor location (17). Tumors >50 ml in size and those associated with the central airways also are associated with greater frequencies of radiation pneumonitis (26). Some investigators find that the incidence of severe radiation toxicity (including a decrease in pulmonary function, pneumonia and pleural and pericardial effusions) is 60% greater for central tumors compared with peripheral ones (27). The unique metastatic characteristics of OS, including affinity for the lung, usually small lesions and often location at peripheral parts of the lung, may make SRS as a potential therapy for this disease. Until now, there are few data in the literature on the role of SRS in pulmonary metastases from OS.

The stereotactic gamma-ray body therapeutic system, or body gamma knife, is developed as a new technology by the OUR International Technology & Science Co. Ltd. (Shenzhen, China). The body gamma knife uses a stereotactic body frame and 30 Co^60^ sources scattered throughout the cavity of the primary chambers. Combination of rotating multiple beam angles with different sources (collimator) and stereotactic body frames can result in sharp dose gradients, high-precision localization, and a high dose per fraction in extracranial locations. This approach delivers a very high biological effective dose (BED) to the center of the target while delivering a microscopic dose to the clinical target volume and a minimized dose to normal tissues. Previous literature from China reported in early stage non-small cell lung cancer, body gamma knife could achieve promising local control and survival with minimal toxicity (28,29).

Thus, we reviewed the clinical data of OS patients suffered from pulmonary metastases treated with body gamma knife in our hospital (Shanghai), and compare the efficacy with that of surgical resection to determine if SRS could achieve acceptable local control and survival in this subset of OS patients.

## Materials and methods

From January 2005 to December 2012, patients with nonmetastatic OS of the extremity were diagnosed and treated at our institution in Shanghai, according to four different neoadjuvant protocols of chemotherapy reported in detail in previous studies (30–34). Diagnosis of OS, established by clinical and radiological findings, was always confirmed on histologic slides of tumor tissue obtained from an open or trocar biopsy and from the resected specimens.

A complete medical history was obtained for all patients, who also underwent a thorough physical examination and several chemical laboratory tests. The primary tumor was evaluated on standard radiographs, and in the most recent cases, also by MRI. The absence or presence of bone metastases was ascertained by total body scans, whereas chest CT scans of the chest were used to exclude lung metastases. Surgery consisted of amputation or limb salvage. During postoperative chemotherapy, besides the clinical evaluation, patients were checked every two months with CT scans of the chest and treated limb. After completion of adjuvant chemotherapy, patients were followed in the outpatient clinic with CT every two months for two years, every three months in the 3rd year and then every six months.

### Patient selection after relapse to enter this review

All the patients who relapsed with only pulmonary metastasis during the period of postoperative chemotherapy or follow-up were eligible for this study since it was designed to precisely evaluate the efficiency of SRS on pulmonary metastasis. After relapse with pulmonary metastasis, all patients were treated at our institution. In chest CT scan, the number of pulmonary metastasis was defined from consensus among at least 3 radiological oncologists.

### Treatment for pulmonary metastasis: surgical resection

For patients who received surgical resection for pulmonary metastasis. Criteria for resectability of lung metastases were: i) no pleural or pericardial effusions; ii) no metastases in other organs besides lungs; and iii) complete resectability leaving adequate residual pulmonary functions (by removing all evident metastatic lesions with no tumor tissue at the resection margins). They underwent a monolateral or bilateral thoracotomy at the same time. If found to be necessary during surgery, wedge resections were performed manually using vascular clamps and resorbable sutures. A lobectomy or a pneumonectomy was performed when necessary depending on the extension, number and site of the pulmonary lesions. Dissection of hilar lymph nodes was performed when necessary. After resection, all the specimens were checked by histological examination.

### Radiotherapy equipment

The body gamma knife uses rotary conical surface focusing to focalize 30 Co^60^ sources with total activity of 8500 Ci, the focal dose rate at the initial source setting was 3 Gy/min. The body gamma knife consists of a radiation source, collimator and treatment bed. The head of radiation source is an iron ball rind with 30 Co^60^ sources scattered throughout the cavity of the primary collimator. The source body rotates horizontally around the central axis with the 30 bundles of gamma ray directed toward a focal target. In the present study, three chamber groups of with collimator aperture diameters of 3, 12 and 18 mm, respectively, were used; the full width at half-height of the dose-field range at the target was 10, 30 and 50 mm, respectively. As the aperture diameter of the collimator decreased, the density of the distributed dose increased, and the periphery dose decreased. Three groups of terminal collimators with different apertures direct the focusing of the radials. Target volume of 1–10 cm in diameter could be treated using a combination of collimators with different aperture diameters. The treatment bed can move in X, Y and Z directions and can automatically adjust the target to the focal point of the radials.

### SRS planning and delivery

Supine or prone position was selected according to CT scan. Patients were immobilized using a vacuum bag covering them from the head to the pelvis. Each patient underwent slow CT simulation at 10 sec/slide with a CT-slide thickness of 5 mm and CT-slide interval of 5 mm to take into consideration tumor motion. Selected patients with significant tumor motion (>1 cm) were evaluated fluoroscopically. Additional margins for tumor motion were added based on the results of the fluoroscopic analysis. After the scan was finished, the positional parameters were recorded in order to repeat the position when the patient was irradiated. The images of CT simulation were then imported into the treatment planning system (OUR WB-GR TPS99). Reconstructions were performed on a three-dimensional conformal radiotherapy planning algorithm.

The gross target volume (GTV) was delineated in the lung window, and a minimum margin of 1 cm was used to form the planning tumor volume (PTV) from the GTV. Either a single focus or multiple foci of radiation beams were used based on the size of the PTV. For example, if treatment of a round <3 cm PTV was required, a single focus of radiation beams using collimators with an aperture diameter of 12 mm was used. However, if treatment of a >3–10 cm PTV was required, a combination of multiple foci of radiation beams using different collimators with different aperture diameters depending on the shape of the PTV was applied to ensure conformal radiotherapy.

Until now, there are few data on the proper radiation dosage of SRS in treating pulmonary metastases from OS. Therefore, we used the radiation dosage reported by Xia *et al* (28) as a reference, considering the similarity of the location of pulmonary lesions and size (most were periphery and <3 cm). A radiation dose of 50 Gy was prescribed to the 50% isodose line covering at least 95% of the PTV (5 Gy/fraction). In addition, delivery of a total dose of 70 Gy (70% isodose line) covering at least 90% of the GTV (7 Gy/fraction) was required. Three-dimensional imaging of isodose coverage of GTV and PTV was used to select aperture diameter, and number and location of target foci depending on the size and shape of the target volume. Radiotherapy was delivered over 2 weeks in 5 fractions per week. In general, the volume of the lung receiving at least 20 Gy was required to be <20%. Moreover, the dose delivered to critical structures such as the main bronchi, esophagus, trachea, heart and major blood vessels was required to be <50 Gy (5 Gy/fraction), and the dose delivered to the spinal cord was required to be <30 Gy (3 Gy/fraction) (28).

### Data collection and assessments

Patients were evaluated through physical examination and routine laboratory analyses (blood count, renal and liver functions) every 2 weeks. Radiologic investigations were performed at every 2 month until progress (assessed by RECIST 1.1).

The aim of this study was to compare efficiency of SRS on pulmonary metastasis with that of surgical resection. The primary endpoint of the study was post-relapse progress-free survival (PRPFS), which was calculated from the date of pulmonary metastasis until progress or last follow-up (assessed by RECIST 1.1). The secondary endpoint was post-relapse overall survival (PROS), which was calculated from the date of pulmonary metastasis until death or last follow-up. Toxicity was assessed according to the National Cancer Institute Common Toxicity Criteria (V3.0). The radiation reaction was classified as early or late side effects according to Radiation Therapy Oncology Group toxicity criteria.

Ethics approval for the study was provided by the independent ethics committee, Sixth People’s Hospital, Shanghai JiaoTong University. Informed and written consents were obtained from all patients or their advisers according to ethics committee guidelines.

### Statistics

The evaluation of PRPFS and PROS was performed using the Kaplan-Meier method for calculating survival curves. Differences among the SRS and surgical-resection groups were compared by means of the χ^2^ test and t-test. Multivariate analyses of survival including the variables that correlated with PRPFS and PROS (i.e., interval from to pulmonary metastasis, number of lesions, monolateral or bilateral lungs) were carried out using the Cox proportional hazards model. The significance was defined at a 2-sided, P-value of <0.05. Statistical analysis was performed using the SPSS software, version 13.0 (SPSS Inc., Chicago, IL, USA).

## Results

### Characteristics of patient and pulmonary metastasis at relapse

Fifty-eight patients were eligible for this retrospective review. At initial diagnosis, the median age of all 58 patients was 21 years (range, 8–59 years), among them, there were 41 males (70.7%) and 17 females (29.3%), 26 received amputation (44.8%) while the other 32 (55.2%) received limb salvage, the initial tumor sites were 33 at femur (56.8%), 19 at tibia (32.7%), 5 at humerus (8.6%) and 1 at fibula (1.7%).

The median interval from the start of treatment to pulmonary metastasis was 12.4 months (range, 2–70.7). Among the 58 patients with pulmonary metastasis, 13 relapsed after 24 months (22.4%), 17 relapsed within 12–24 months (29.3%), 28 relapsed within 12 months (48.3%). Twenty-nine patients relapsed with monolateral lesions (50%) while the other half with bilateral lesions. Twenty-seven (46.5%) patients relapsed with only one nodule while 31 (53.4%) patients relapsed with two nodules or more. The number of nodules are defined by CT scans of the chest before onset of treatment for relapse.

The number of pulmonary lesions in 58 patients range from 1 to 9, median 2 lesions. From CT scan, number of pulmonary lesions in the SRS group (range from 1 to 9 lesions, mean 2.19±1.79) was parallel to (P>0.05) that in the surgical group (range from 1 to 5 lesions, mean 2.59±1.44).

### Treatment of relapse

Of the patients 31 underwent surgical resection (surgical group, consist of 14 wedge resections and 17 lobectomies) and 27 underwent gamma knife SRS (SRS group). For patients in surgical group, radiologically metastatic nodules were all proven to be metastasis from OS histologically after resection. The characteristics of the patients in two groups are listed in Table I. Factors such as age, gender, initial tumor site, histotype of tumor, method of surgery for initial tumor, time to relapse were well balanced between the groups. No difference was observed in aspects of pulmonary lesion site and pulmonary lesion number between surgical group and SRS group.

### Outcome

Until last follow-up in October 2013, the follow-up time after the treatment for relapse in surgical and SRS group was 5–96 months (median, 22 months) and 8–50 months (median, 18 months), respectively. In patients without progression or still alive after treatment for relapse, their minimum follow-up time had reached 2 years.

**Figure 1.**
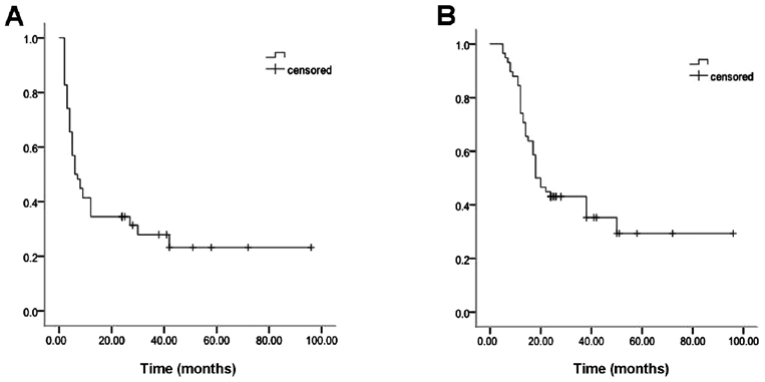
Kaplan-Meier plots of post-relapse progress-free survival (A) and post-relapse overall survival (B) for all patients.

Of 58 patients, the median time of PRPFS and PROS was 6 months (range, 2–96 months) and 18 months (range, 5–96 months), respectively (Fig. 1A and B). Until last follow-up in October 2013, there were 15 relapses with pulmonary metastasis, 3 with bone metastasis, 1 with pulmonary and bone metastasis in surgical group. In the SRS group, there were 4 relapses with local pulmonary recurrences, 13 with pulmonary metastasis, 2 with bone metastasis, 1 with pulmonary and bone metastasis, and 1 with local recurrence at initial tumor site.

**Figure 2.**
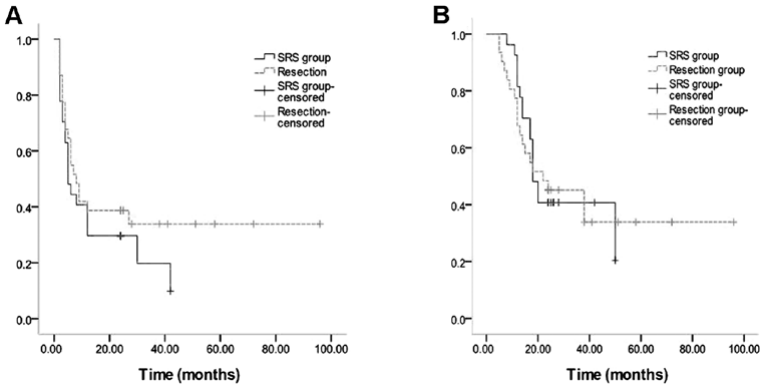
Kaplan-Meier plots of post-relapse progress-free survival (A) and post-relapse overall survival (B) for the surgical and SRS group. The median time of PRPFS and PROS in SRS group was parallel to that in the surgical group.

Four months after completion of SRS, the CR rate was 59.2% (16/27), the PR rate was 11.1% (3/27), the disease control rate was 70.3%. In the surgical group, 4 months after operation, the CR rate was 70.9% (22/31), the disease control rate was also 70.9% (No PR in surgical resection). The two-year progression-free survival rate was 38.7% (12 CRs, 9/31) in the surgical group and 33.3% (8 CRs and 1 PR, 9/27) in the SRS group. The two-year survival rate was 48.3% (15/31) in the surgical group and 40.7% (11/27) in the SRS group. The median time of PRPFS in the surgical and SRS group was 8 and 5 months, respectively. The median time of PROS in the surgical and SRS group was 22 and 18 months, respectively (Fig. 2A and B). Differences in median time of PRPFS, median time of PROS, two-year progression-free survival rate and two-year survival rate between two groups were not significant (P>0.05).

Cox proportional hazards model indicated that time to relapse and number of pulmonary lesions are associated significantly with PRPFS and PROS. Moreover, these two factors were well balanced between the groups (Table I).

### Adverse events for SRS and surgical resection

**Figure 3.**
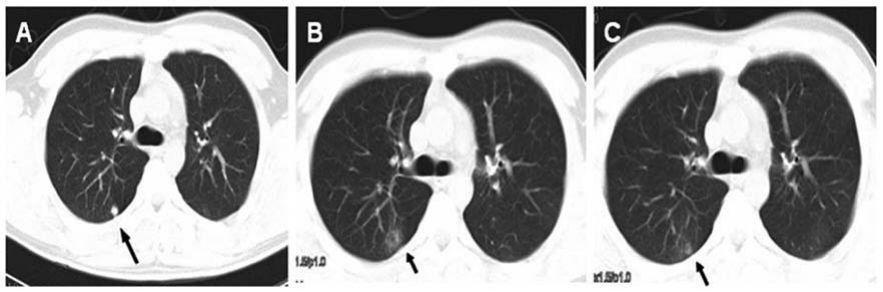
Radiation pneumonitis occurs 2 months after SRS treatment. Before SRS treatment (A). Two months after SRS treatment (B), CT scan showed radiation pneumonitis (black arrow) without any complaint from the patient. (C) Without medical intervention, radiation pneumonitis resolved two months later.

Acute toxicity was defined as events occurring in the first 3 months after SRS treatment, and late toxicity was defined as events occurring >3 months after SRS treatment. Generally speaking, the addition of SRS for patients was well tolerated. There have been no cases of grade 3–5 toxicity or possible treatment-related death observed. The most common acute toxicity from SRS was grade 1–2 pneumonitis in 9 patients (7 patients with grade 1 while 2 patients with grade 2), account for 33.3%. All cases of radiation pneumonitis occurred 1–2 months after treatment and resolved within 1–2 months (Fig. 3). We did not observe other complications include the development of pleural effusions, hemoptysis, tracheoesophageal fistula, pericardial effusions, or pneumothoraces. In terms of late toxicity, until the last follow-up at October 2013, there was no incidence of radiation pulmonary fibrosis or grade 3–5 radiation pneumonitis.

**Figure 4.**
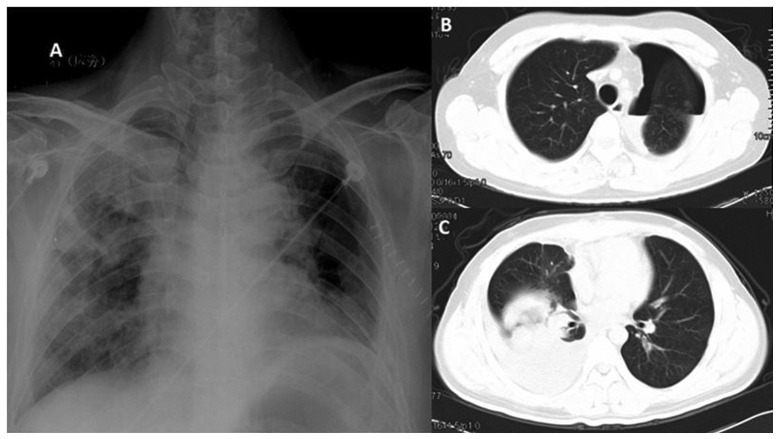
Complications after surgical resection treatment. (A) Pneumonitis. (B) Persistent pneumothorax. (C) Intrapulmonary hematomas accompanied with blood pleural effusion.

**Figure 5.**
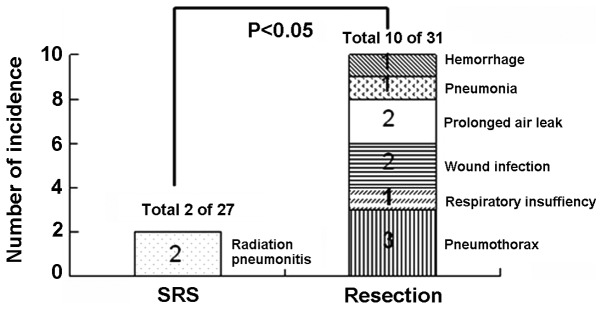
Incidence of complications which were resolved by medical intervention in SRS and resection groups. There were more patients with complications needing medical intervention (10 out of 31) in the surgical group than (2 out of 27) in the SRS group.

For the 31 patients who underwent surgical resection, there were no surgery-related deaths. The most common complication is persistent pneumothorax in 3 patients. These pneumothorax resolved by keeping the drainage *in situ* for a longer period of time (10–15 days) and by placing a unidirectional valve removed once the pneumothorax resolved. Other complications were respiratory insufficiency for 1 patient, superficial wound infections for 2, prolonged air leak for 2, pneumonia for 1, hemorrhage for 1 (Fig. 4). With aggressive medical intervention, all the 10 patients recovered within 2 months. More patients with complications needing medical intervention (10 out of 31) was recorded in the surgical group than that (2 out of 27) in the SRS group (P<0.05, Fig. 5).

## Discussion

In the past, 80–90% of patients with osteosarcoma (OS) of the extremities treated by surgery alone, mostly amputation, relapsed in the first 12 months (3). In the last 25 years, adjuvant and neoadjuvant chemotherapy have greatly improved prognosis of these patients. However, still 30–40% will relapse. Lung is the most frequent metastatic site. Of 570 patients with OS of the extremities treated at the Istituto Ortopedico Rizzoli from 1983–1995, 206 (36%) relapsed only in the lung (35). In addition, nearly 40% of patients who had undergone complete remission of lung metastases relapsed in the lung (11). Although advances in chemotherapeutic strategy and surgical approach have significantly improved the prognosis of these patients (9–16), optimal treatment strategy is still undefined.

The advances of technology in radiation therapy have enabled stereotactic radiosurgery (SRS) as a novel technique for lung cancer. SRS allows the delivery of a higher dose of radiation to the tumor while reducing the irradiation of the surrounding normal tissue. Previous studies have shown that SRS was feasible and efficient in patients with primary and secondary pulmonary tumors, including pulmonary metastasis from soft tissue sarcoma (24). Moreover, body gamma knife, developed as a new technology in China, has showed its comparable efficacy with surgical resection in early stage NSCLC (28,29). In addition, some patients in China are unwilling to undergo surgery because of traditional cultural customs, and they are more willing to choose SRS, which further makes body gamma knife a promising treatment.

In our study, for the first time, we found that the delivery of SRS treatment by body gamma knife on OS patients with pulmonary metastasis could yield an outcome which was equivalent to that from surgical resection. Although there was a 3 months difference in PRPRS and a 4 months difference in PROS with better results for the surgical group, the difference is not significant. We believed that it may be contributed to a higher proportion of patients with one pulmonary metastatic lesion in the surgical group (17 out of 31, while 10 out of 27 in the SRS group), as Cox proportional hazards model indicated that number of pulmonary lesions was a key factor which could affect PRPFS and PROS. It is reasonable for us to believe that SRS treatment might have an opportunity to play a role in pulmonary metastasis from OS as it has played in intracranial neoplasms.

A major limitation of the body gamma knife is that the isodose distribution is mainly circuital or elliptical. If the tumor shape is anomalous, obtaining satisfactory conformity of the isodose line is difficult. In OS, the unique distribution pattern of pulmonary metastatic lesion (small, peripheral and round lesions) makes this limitation unobvious. Besides, consideration of tumor motion is critical in SRS in a patient whose tumor moves significantly during radiotherapy. A recent study used four-dimensional CT to investigate patients with lung cancer and found that the tumor moved >1 cm during breathing in 13% of the patients, particularly those with small lower lobe tumors close to the diaphragm (36), suggesting an individualized tumor-motion margin should be considered for such patients. In our study, slow CT simulation and fluoroscopy were used to compensate for tumor motion during breathing. We found 8 patients in SRS group had significant tumor motion (>1 cm). Although 4 patients with local pulmonary recurrences were well balanced in patients with significant tumor motion (2 out of 8) or not (2 out of 19), we could not exclude the influence by small number of local pulmonary recurrences which might limit statistical power to show the difference. In addition, we believe better evaluation and targeting of tumor motion using improved on-board imaging may allow further dose escalation and reduce the risk of local recurrence.

In general, the most common acute toxicity from SRS are pneumonitis and pneumothoraces, other side effects include pleural effusions, hemoptysis, tracheoesophageal fistula and pericardial effusions. Some of these side effects might be life-threatening. The most common later toxicity from SRS are fibrosis and grade 3 to 5 radiation pneumonitis. Encouragingly, the toxicity of body gamma-knife radiosurgery in our study was well tolerated. The 27 patients who underwent SRS experienced no treatment-related death or serious side effects, and more inspiring, the SRS group needed less medical intervention to resolve side effects than the resection group, due to only 9 patients with grades 1–2 pneumonitis. The safety of SRS on pulmonary metastasis from OS may be contributed to not only the technical improvements but also the unique metastatic characteristics of OS, which are usually small lesions and often located at peripheral parts of the lung. For SRS, smaller lesions at peripheral site of the lung without association with central airways means less possibility of lung injury (22,23), which implies that pulmonary metastasis from OS is a potentially good candidate for SRS.

There are several shortcomings in our study. First, our study was limited to its retrospective nature with possible patient selection bias which could affect the prognosis. In China, patients have to pay for SRS treatment as it is not covered by medical insurance. This might make some patients eligible for SRS treatment to choose surgery. Secondly, due to the limited number of patients, we believed the efficiency of SRS treatment on OS patients with pulmonary metastasis, compared with that of resection, is still far from being defined. Thirdly, because our study is the first to investigate the efficacy of body gamma knife in treatment of pulmonary metastasis from OS, we took the prescribed radiation dosage reported by Xia *et al* (28), which investigated NSCLC, as a reference. The radiation dosage might not be optimal.

SRS is a potentially alternative treatment for pulmonary metastasis of OS after the failure of adjuvant chemotherapy, especially for those patients who were medically unfit for a resection (on the basis of age, comorbidities), or who refused surgery. Further studies to confirm the results, especially prospective clinical trials, focusing on the efficiency of SRS treatments for OS patients with pulmonary metastasis compared with surgical resection, should be strongly considered.

## Figures and Tables

**Table I.** Characteristics of patients wth occurrence of pulmonary metastasis.

Demographic data	Resection	SRS	P-value
No. of subjects	31	27	
Gender			0.96
Male	22	19	
Female	9	8	
Age (years)			
>18	22	17	0.51
≤18	9	10	
Site			
Femur	18	15	0.84
Tibia	10	9	
Humerus	2	3	
Fibula	1	0	
Surgery			
Amputation	15	11	0.56
Limb salvage	16	16	
Histotype			
Classic	29	24	0.87
Others	2	3	
Site of pulmonary lesions			
Monolateral	19	10	0.11
Bilateral	12	17	
No. of pulmonary lesions			
Solitary	17	10	0.17
Multiple lesions (≥2)	14	17	
Time to relapse (years)			
≤2	22	23	0.20
>2	9	4	
